# Fabrication of 3D binder-free graphene NiO electrode for highly stable supercapattery

**DOI:** 10.1038/s41598-020-68067-2

**Published:** 2020-07-08

**Authors:** Elochukwu Stephen Agudosi, Ezzat Chan Abdullah, Arshid Numan, Nabisab Mujawar Mubarak, Siti Rahmah Aid, Raúl Benages-Vilau, Pedro Gómez-Romero, Mohammad Khalid, Nurizan Omar

**Affiliations:** 1 Department of Chemical Process Engineering, Malaysia-Japan International Institute of Technology (MJIIT), Universiti Teknologi Malaysia (UTM), Jalan Sultan Yahya Petra, 54100 Kuala Lumpur, Malaysia; 2 State Key Laboratory of ASIC and System, SIST, Fudan University, Shanghai, 200433 China; 3 Department of Chemical Engineering, Faculty of Engineering and Science, Curtin University, 98009 Sarawak, Malaysia; 4 Department of Electronic Systems Engineering, Malaysia-Japan International Institute of Technology (MJIIT), Universiti Teknologi Malaysia (UTM), Jalan Sultan Yahya Petra, 54100 Kuala Lumpur, Malaysia; 5 Catalan Institute of Nanoscience and Nanotechnology (ICN2), CSIC and The Barcelona Institute of Science and Technology, Campus UAB, 08193 Bellaterra, Barcelona Spain; 6 Graphene & Advanced 2D Materials Research Group (GAMRG), School of Science and Technology, Sunway University, Subang Jaya, 47500 Selangor, Malaysia; 7 Department of Gigaphoton Next GLP, Graduate School of Information Science and Electrical Engineering, Kyushu University, Fukuoka, 819-0395 Japan

**Keywords:** Environmental social sciences, Materials science, Nanoscience and technology

## Abstract

Electrochemical stability of energy storage devices is one of their major concerns. Polymeric binders are generally used to enhance the stability of the electrode, but the electrochemical performance of the device is compromised due to the poor conductivity of the binders. Herein, 3D binder-free electrode based on nickel oxide deposited on graphene (G-NiO) was fabricated by a simple two-step method. First, graphene was deposited on nickel foam via atmospheric pressure chemical vapour deposition followed by electrodeposition of NiO. The structural and morphological analyses of the fabricated G-NiO electrode were conducted through Raman spectroscopy, X-ray diffraction (XRD), field emission scanning electron microscopy (FESEM), and energy dispersive X-ray spectroscopy (EDS). XRD and Raman results confirmed the successful growth of high-quality graphene on nickel foam. FESEM images revealed the sheet and urchin-like morphology of the graphene and NiO, respectively. The electrochemical performance of the fabricated electrode was evaluated through cyclic voltammetry (CV), galvanostatic charge–discharge (GCD), and electrochemical impedance spectroscopy (EIS) in aqueous solution at room temperature. The G-NiO binder-free electrode exhibited a specific capacity of ≈ 243 C g^−1^ at 3 mV s^−1^ in a three-electrode cell. A two-electrode configuration of G-NiO//activated charcoal was fabricated to form a hybrid device (supercapattery) that operated in a stable potential window of 1.4 V. The energy density and power density of the asymmetric device measured at a current density of 0.2 A g^−1^ were estimated to be 47.3 W h kg^−1^ and 140 W kg^−1^, respectively. Additionally, the fabricated supercapattery showed high cyclic stability with 98.7% retention of specific capacity after 5,000 cycles. Thus, the proposed fabrication technique is highly suitable for large scale production of highly stable and binder-free electrodes for electrochemical energy storage devices.

## Introduction

The evolution of electronics has catalysed the demand for energy storage devices. On the other hand, the rapid depletion of fossil fuels and their detrimental environmental effects led to the development of renewable energy resources and energy storage devices^1,2^. Fuel cells, supercapacitors, and second generation lithium-ion batteries are promising and sustainable energy storage systems that have attracted huge research interest due to their high energy and power densities, long cycle life, and eco-friendliness^3–6^. Notably, batteries and supercapacitors are adjudged to be the major energy storage systems. In this context, developing novel devices capable of storing harvested energy from renewable energy sources are desirable. Batteries can gain high energy density based on the redox reactions at their electrodes but are flawed by insufficient power density^7^. Hence, supercapacitors (SCs) have gained credence due to their capabilities to deliver high power density, although they are limited by low energy density^8^. In addition, SCs have fast charge–discharge capability and prolonged cyclic efficiency, among other advantages for ecological suitability^9^. Therefore, numerous investigations are underway geared at enhancing the energy density of SCs without compromising their high power capability. Nowadays, the idea of developing hybrid electrical energy storage devices to bridge the gap between the parametric quantities of batteries and SCs has been sought. This hybrid device is termed *‘supercapattery’*. Supercapattery employs the high efficiency battery-type electrode materials, which breaks through the slow kinetics, low rate performance, and low cycling stability^10,11^. Supercapattery aggregates the high energy potential of the battery-grade materials (redox-active materials enabling faradaic reaction processes) as a positive electrode (high energy density) and carbonaceous materials as a negative electrode (high power density)^12,13^.

Generally, carbonaceous materials are used as EDLC electrodes due to their low cost, high surface-area-to-volume ratio, high electrical conductivity, and high thermal stability. Carbonaceous materials store charges at electrode/electrolyte interface by physical adsorption, which renders high power density and long cycle life^14^. In contrast, battery-grade materials employ redox reactions, which leads to high energy density and high rate capability^15^. Generally, metal oxides such as Fe_2_O_3_, MnO_2_, NiO, MoO_2_, Co_3_O_4,_ etc. are redox-active and have been utilized as positive electrode materials for achieving high electrochemical energy storage^16,17^. NiO, as a redox transition metal active material, has high thermal and chemical stability, high theoretical specific capacity, low price, abundance, and eco-friendly^16^. Thus, it is highly desirable to combine NiO with a highly conductive platform to achieve an enhanced electroactive positive electrode.

Graphene, an allotrope of carbon, has intriguing properties such as excellent electrical and thermal conductivity (3,000–5,000 W m^−1^ K^−1^), a large surface-area-to-volume ratio (2,600–2,620 m^2^ g^−1^), fast electron mobility, and superior mechanical strength (Young’s modulus of ≈ 1 TPa)^18^. These qualities make graphene an ideal candidate to be utilised as the conductive platform that increases electrochemical surface area, prevents nanoparticle agglomeration, and, therefore, enhances cyclic stability. By combining battery-grade metal oxide(s) with graphene, the electrochemical properties of the composite can be enhanced, and thus, the specific capacity of the device can be improved significantly^19–22^. Therefore, graphene is an ideal building block of electrode material to achieve high specific capacity, rapid charge–discharge kinetics, excellent rate capability, and cyclic stability in electrochemical energy storage devices^23^. The 3D carbon nanostructured skeleton offers a larger surface area, and hence provides extended growth sites for transition metal oxides (TMOs)^24^. A well-decorated carbon material with TMOs facilitates rapid charge transfer and achieves high rate capability^25–27^.

Typically, polymeric binders are used for the fabrication of electrodes for energy storage devices. However, electrode conductivity is significantly degraded due to the introduction of low conductivity polymeric binders, resulting in the poor electrochemical performance of the device. High rate capability and excellent cycle life can be achieved with a binder-free electrode due to the faster charge and ion transport^28–30^. All electro-active materials fabricated for faradaic charge storage undergo repeated ingression and egression of ions, which results in fatigue. However, the effective way to mitigate this inherent problem is to support the active material as a thin coating onto electrochemically inert nanomaterials to achieve high cycling stability^31,32^. Electrodeposition is an effective technique that is used for the direct growth of metal oxide thin films on graphene. The electrodeposition technique is appropriate in reducing the amount, size, and weight as well as the electrolyte (ionic) resistance of electrode materials. The weight and the thickness of the electro-active materials can be controlled by adjusting the electrodeposition parameters such as the concentration of precursor solutions, time, pH, deposition potential, etc.^33,34^.

In view of the above discussion, a 3D binder-free graphene electrode is fabricated by CVD followed by electrodeposition of NiO thin film for energy storage application. Binder-free electrodes are desirable due to the low cost, lightweight, low contact resistance, and offers rapid electrode fabrication, unlike electrodes with binders. Moreover, the low specific capacity and insulating nature of binders cause high contact resistance and significantly degrades the conductivity of the electrode, resulting in poor electrochemical performance of the energy storage device/system^35^. Although, some studies have investigated the electrochemical parameters of NiO for energy storage applications^16,36^, the novelty of this study lies in the development of binder-free electrode material through the combination of graphene and NiO thin film as a binary nanocomposite. Graphene provides a highly conductive platform for charge transport, while NiO produces a synergistic effect for enhanced electrochemical energy storage. In the composite, graphene not only aids in the shuttling of electrons but also contributes in charge storage mechanism via the EDLC effect. The developed G-NiO electrode was further employed as a positive electrode for supercapattery fabrication with activated charcoal as the negative electrode. The fabricated supercapattery showed excellent electrochemical performance and outstanding stability. Hence, the proposed method for the preparation of the binder-free electrode can open new avenues for the development of high-performance supercapattery, which can have commercial value.

## Results and Discussion

### Structural and morphological characterisations

**Figure 1:**
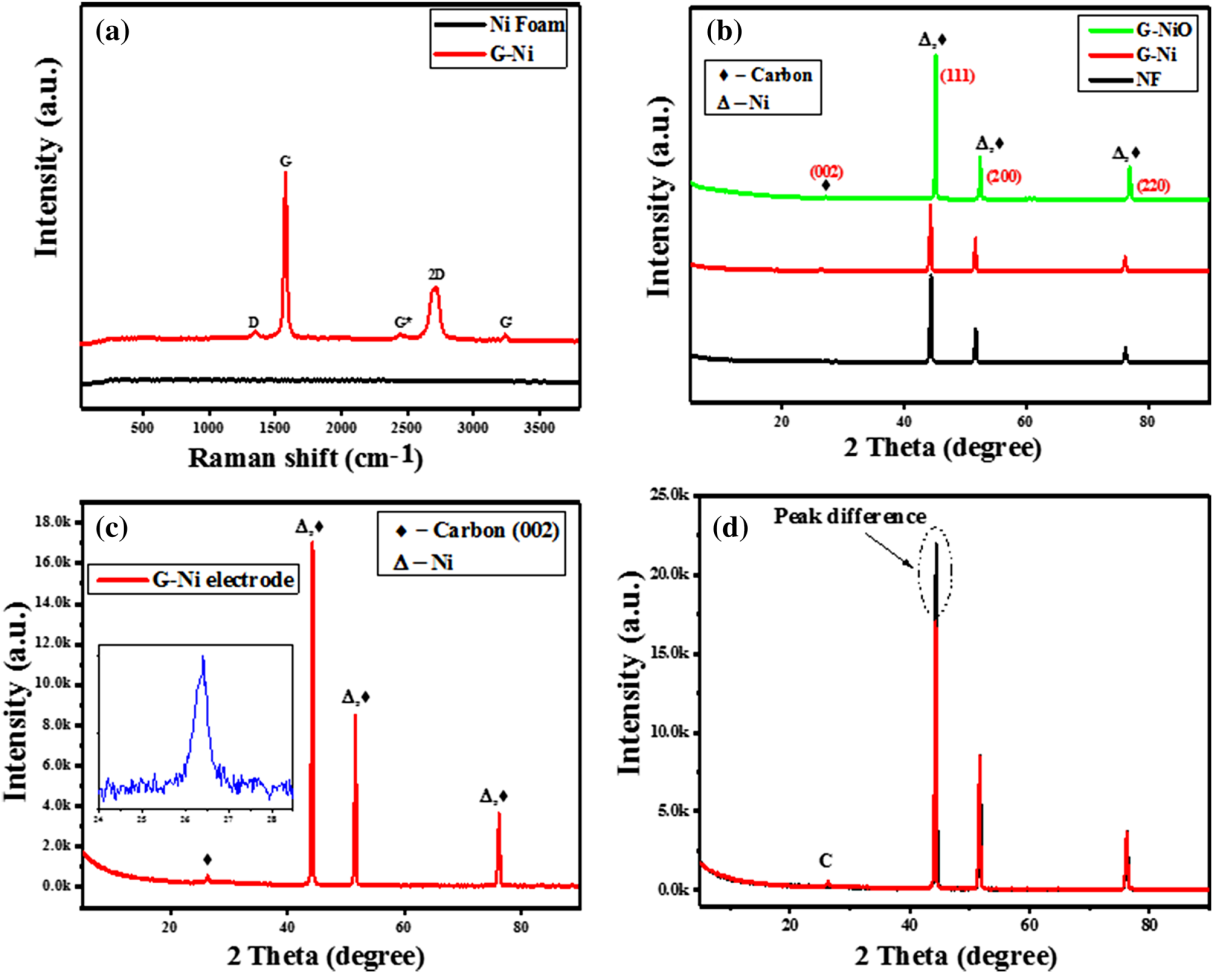
Comparative Raman spectra of (**a**) Ni foam and G-Ni electrode, (**b**) comparative XRD patterns of Ni foam, G-Ni and G-NiO, (**c**) XRD pattern of G-Ni electrode and the inset depicts the expanded peak position of graphene, and (**d**) overlay of the XRD patterns of NF and G-Ni showing the peak difference.

Raman spectrum gives useful information on the quality of the as-synthesized graphene and number of layers. These properties are typically estimated based on the ratio of *I*_2D_/*I*_G_ band intensities as well as a shift in their peak positions. The comparative Raman spectra of the bare nickel foam (NF) and G-Ni electrode are shown in Fig. 1a. Bare NF maintained a straight line without any peaks. However, the G-Ni electrode showed the prominent graphitic characteristics of the *sp*^2^ hybridized carbon atoms (G and D bands) well bonded on the NF (Fig. 1a). This is an evidence of highly crystalline, few to multi-layered graphene sheets deposited on the NF. The graphitic layers were observed at the wavenumber value of 1,380 cm^−1^, 1577 cm^−1^, and 2,720 cm^−1^ for D, G, and 2D bands, respectively. The D band showed a slightly defective graphene structure, while the G and 2D bands depicted the first (tangential stretching mode of highly oriented pyrolytic graphite—HOPG) and second-order phonon scattering, respectively. These results agree with the previous studies^37–40^.

X-ray diffraction determines the crystallographic structure of the electrodes. The X-ray diffractometer obtained the XRD patterns over the 2θ range from 5° to 90° with monochromatized Cu K-α radiation. Figure 1b shows the comparative XRD patterns of NF (with lattice parameters of (111) at 44.51°, (200) at 51.85°, and (220) at 76.37° for NF^41^ (which served as baseline spectrum), G-Ni electrode and G-NiO electrode. All the diffraction peaks for Ni and NiO can be indexed as a cubical crystalline phase of NiO (JCPDS card no. 22-1189)^42^. In the NF deposited graphene, a distinct peak was observed at angle 2θ peak position 26.45°, which confirmed the presence of graphene^37,38^. Figure 1c shows the XRD pattern of the G-Ni electrode; inset depicts the expanded graphene peak, which corresponds to the lattice parameter of carbon (002). There was a reduction in the peak intensity due to the graphene layers on the NF (Fig. 1d). It could be seen that the NiO thin film formed homogenous oxide layers on the G-Ni electrode without any obvious impurities. A different spectra showing the expended angle peak positions and peak intensities in the XRD pattern of the G-Ni electrode are presented in the supplementary data online (Figs. S2, S3 and S4).

**Figure 2:**
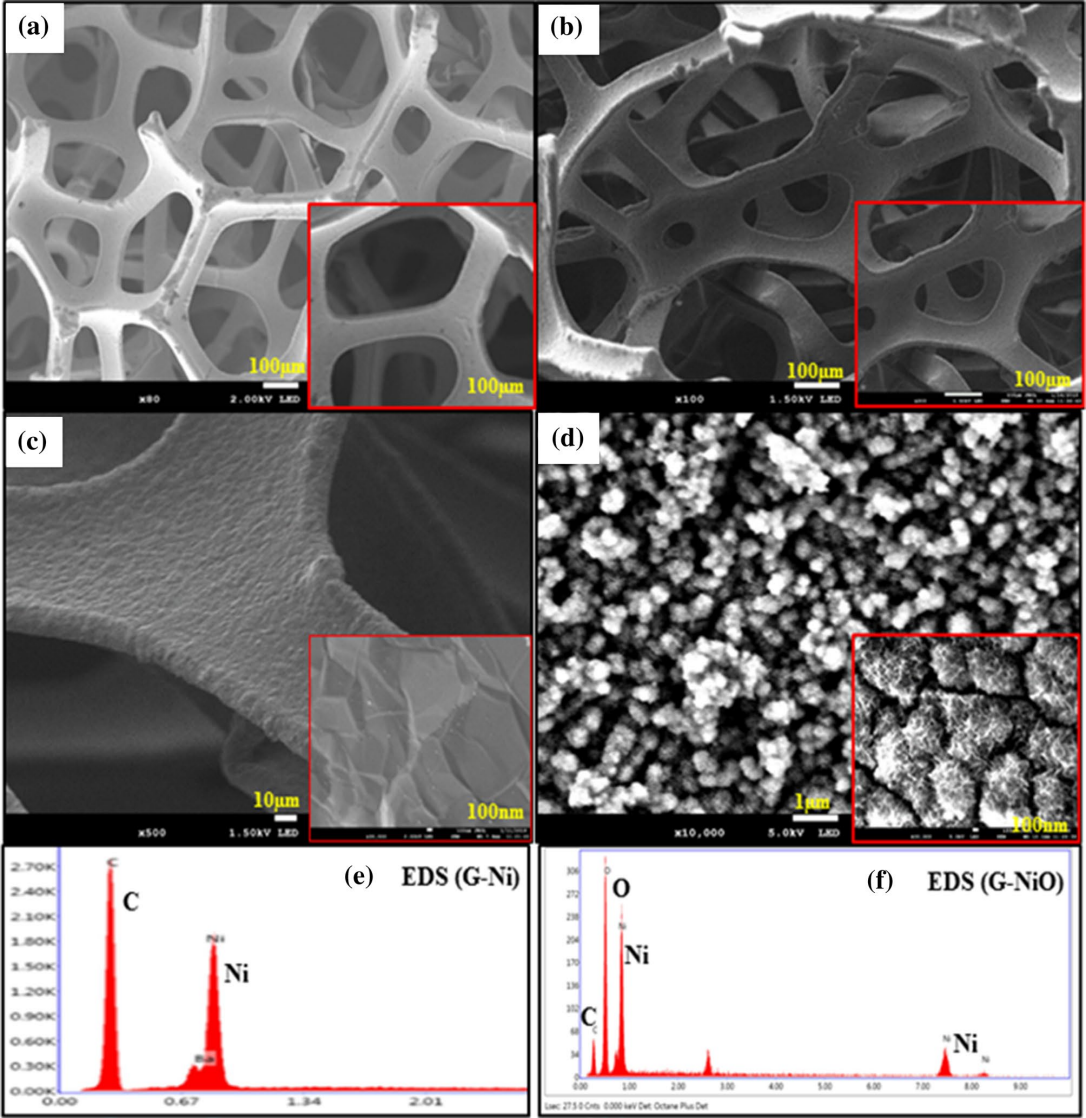
FESEM micrographs of (**a**) Ni foam, (**b**,**c**) G-Ni electrode, (**d**) G-NiO electrode; (**e**) corresponding EDS mapping for G-Ni electrode, and (**f**) corresponding EDS mapping for G-NiO electrode. The insets depict the corresponding high magnification images of the electrodes.

Field emission scanning electron microscopy equipped with energy dispersive X-ray analyser was used to characterize the morphology and elemental composition of the electrodes. Figure 2a shows the FESEM micrographs of NF revealing the clean surface without any contaminations. Similarly, Fig. 2b and Fig. 2c depict the thin film of graphene grown on the NF at different magnifications. The FESEM micrograph of the G-NiO electrode showing the sheet and urchin-like morphology is shown in Fig. 2d. To determine the percentage of the atomic composition for both G-Ni and G-NiO electrodes, EDS mappings were done (Fig. 2e and Fig. 2f) and the results are presented in Table 1. The FESEM image and EDS spectrum of the NiO electrode are provided in the supplementary data online (Fig. S9).

**Table 1:** EDS for G-Ni and G-NiO electrodes.

Electrode	Element	Weight %	Atom %	Net intensity	K ratio
G-Ni	C K	59.46	88.26	1,113.58	0.3414
	Ni K	37.27	11.32	584.67	0.2836
G-NiO	C K	18.22	30.49	20.63	0.0669
	O K	45.44	57.07	112.94	0.2196
	N K	36.34	12.44	23.16	0.3100

### Electrochemical evaluations

**Figure 3:**
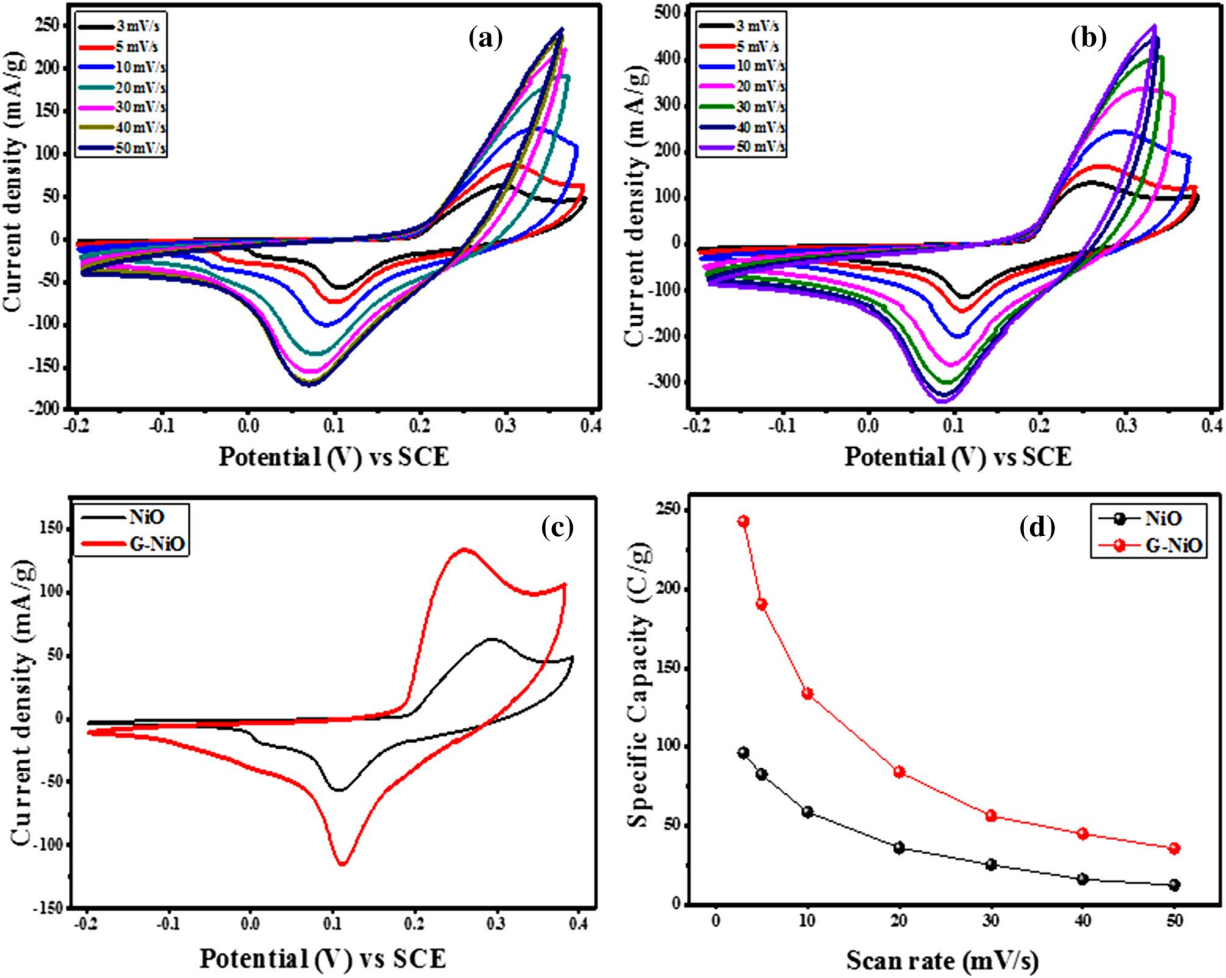
CV curves for (**a**) NiO electrode and (**b**) G-NiO electrode at different scan rates, (**c**) comparative CV curves for NiO and G-NiO at the same scan rate of 3 mV s^−1^, and (**d**) corresponding graphs of specific capacity against scan rates for NiO and G-NiO electrodes.

The cyclic voltammetric responses of NiO and G-NiO electrodes at different scan rates (3–50 mV s^−1^) over the potential window of − 0.2–0.4 V in a standard three-electrode cell are shown in Fig. 3a and Fig. 3b, respectively. The CV curve of the NiO electrode shows well-defined redox peaks, which is an indication of a battery electrode. The peaks indicated the redox transition of Ni ions due to non-capacitive faradaic behaviour^31^. The redox peaks are attributable to the diffusion of electrolyte in the material which suggested that the NiO electrode was showing battery-type behaviour^31,43^. The electrode redox behaviour was based on the Nernstian process as depicted by the peak-shaped CV curves. Distinct redox peaks were observed at high scan rates showing a high rate of capability and good reversibility of the working electrode^44^.

From Fig. 3b, it is evident that, with increased scan rates, the redox peak current of the G-NiO electrode was significantly increased with better reversibility as compared to NiO electrode due to the presence of graphene. The introduction of graphene improved the exposure of active sites of the electrode for the faradaic reactions owing to the enhanced surface area. Additionally, the amorphous phase formed from the graphene and NiO thin film aids electroactivity. Figure 3c illustrates the comparative CV curves of NiO and G-NiO at the same scan rate of 3 mV s^−1^ revealing the higher specific capacity of the latter. G-NiO exhibited a high, and remarkable peak current compared to NiO. It is apparent that the electrochemical performance of the G-NiO was drastically improved, and this was due to the high surface area and conductive platform provided by graphene thin film. This high background current for the G-NiO electrode compared to NiO was as a result of the highly conductive platform of the graphene in the G-NiO electrode. The overlay of CV curves (at 3 mV s^−1^) for the NF and G-Ni electrode showing the significant difference in electrochemical performance is shown in the supplementary data online (Fig. S5). Therefore, the combination of NiO thin film with graphene improved its conductivity and capability performance. The specific capacity values of the electrodes, *Q*_*s*_ (C g^−1^) from the CV curve were calculated from the relation as expressed in Eq. (1).

1 $${Q}_{s}=\frac{1}{v\times m}{\int }_{{V}_{i}}^{{V}_{f}}I \times (V)dV \quad (\text{CV})$$

where *v* and *m* represent the scan rate (mV s^−1^) at which the voltammogram is recorded and the mass loading of active materials (g) respectively. The integral term represents the area under the redox peaks.

The value of the specific capacity as against different scan rates for NiO and G-NiO electrodes are presented in Table S1 of the supplementary data online. The maximum specific capacity achieved from the G-NiO electrode was 243 C g^−1^ which is significantly higher in value as compared to 96 C g^−1^ obtained from NiO electrode. Figure 3d represents the corresponding specific capacity of the two electrodes as a function of scan rate. It could be observed that an increase in the scan rate decreased capacity due to insufficient time for ions to penetrate the inner pores of the electrode, and thus, resulted in a low capacity and vice versa^45^.

**Figure 4:**
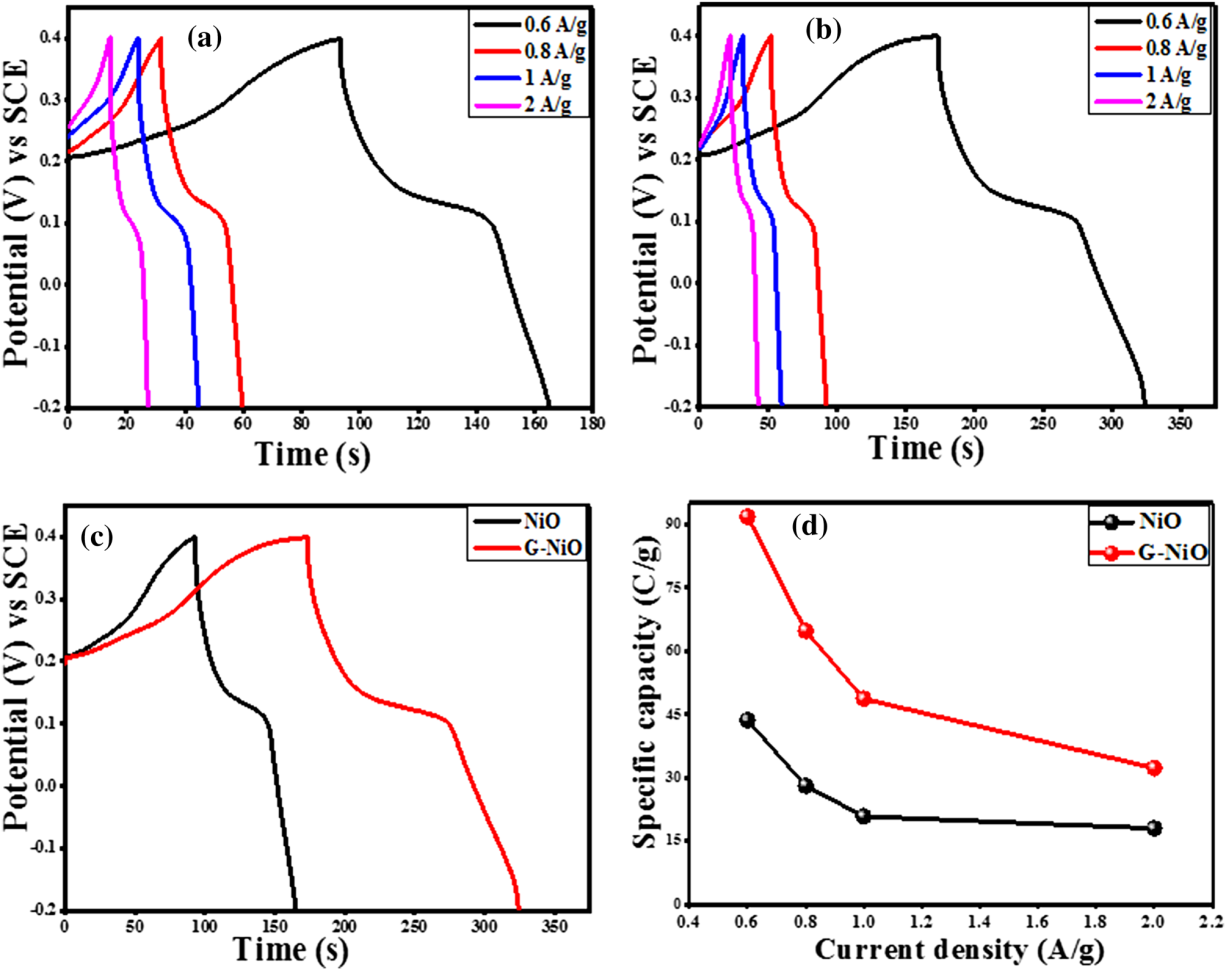
Galvanostatic charge–discharge plots for (**a**) NiO electrode and (**b**) G-NiO electrode at various current densities, (**c**) comparative charge–discharge plots for NiO and G-NiO at the same current density of 0.6 A g^−1^, and (**d**) corresponding graphs of the specific capacity against current density for NiO and G-NiO electrodes.

Similarly, the galvanostatic charge–discharge (GCD) studies were conducted to investigate the stability of the NiO and G-NiO electrodes. Figure 4a and Fig. 4b represent the charge–discharge plots for the two electrodes at different current densities in the range of 0.6 A g^−1^ to 2 A g^−1^ over a potential window of 0.6 V (− 0.2–0.4 V). The battery-like characteristics of the electrodes could be observed from the charge–discharge behaviour which was based on the non-capacitive faradaic reaction mechanism as the electrolyte was dispersed. The discharge time was decreased with an increase in current density which implied an inverse relationship (in both cases). Figure 4c is the comparison GCD plot for both electrodes revealing a higher specific capacity for the G-NiO electrode. The specific capacity of the electrodes, *Q*_*s*_ (C g^−1^) were evaluated from the GCD curve using the relation as given in Eq. (2).

2 $${Q}_{s}=\frac{I \times \Delta t}{m} \quad (\text{GCD})$$

where *I*, *m*, and $\Delta t$ represent the discharging current (*A*), mass loading of active materials (*g*), and time taken to fully discharge the electrode (*s*) respectively. The value of the specific capacity against diverse current densities for NiO and G-NiO electrodes are presented in Table S2 of the supplementary data online.

The specific capacity values were estimated to be 92 C g^−1^ for the G-NiO electrode and 44 C g^−1^ for the NiO electrode. The *Q*_*s*_ value for the G-NiO is significantly higher compared to the results obtained in the previous studies that investigated transition metal oxides (TMOs) as electrodes^46,47^. The GCD measurements showed that the specific capacity value was highest at the lowest current density, and decreased with an increase in current density up to 2 A g^−1^. This effect was well illustrated in the charge–discharge plots where the *Q*_*s*_ value decreased from 92 to 26 C g^−1^ for G-NiO with a retention of 71.2%. Likewise, the NiO electrode retained 58.7% which was a reduction from 44 to 18 C g^−1^. The tremendous enhancement in the specific capacity of the G-NiO electrode is ascribed to the higher specific surface area of graphene (which boosted charge mobility).

It is noted that the 3D nanostructure of the G-NiO electrode enabled excellent acceleration of the ionic diffusion into various directions and thus, minimized differential activation energy of the ionic species. Moreover, the NiO thin film contributed to the synergistic effect of the nanocomposite. The direct relationship of specific capacity with surface area applies to the related studies involving mesoporous materials such as NiO^48^ and carbon^49^, however in the binder state. A larger current accommodation during the charge–discharge process indicates a good battery-grade material behaviour with respect to charge storage mechanism, thereby providing more active sites to generate a larger number of redox activities owing to the large specific surface area. This capability is demonstrated by the longer discharge time as recorded in Fig. 4c for the G-NiO electrode. Figure 4d depicts the comparative specific capacity as a function of current density for the NiO and G-NiO electrodes.

**Figure 5:**
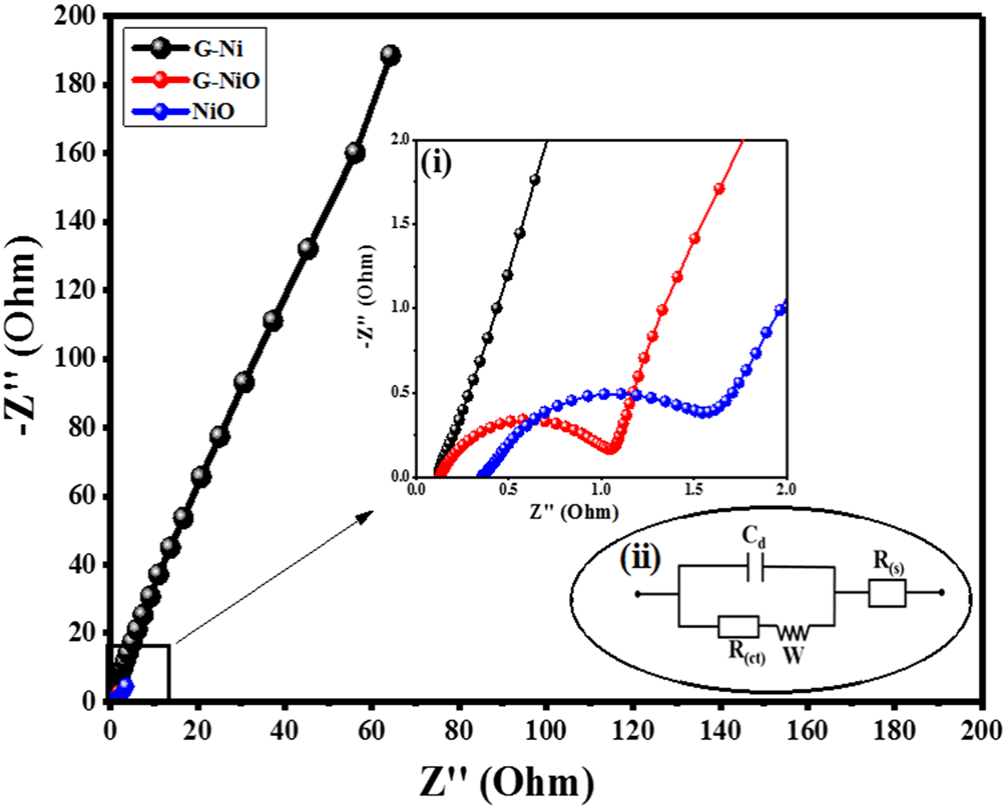
Nyquist plots of G-Ni, NiO, and G-NiO electrode; inset (i) shows the expanded EIS spectra revealing the corresponding charge-transfer resistances and inset (ii) the equivalent represents the circuit diagram for the EIS.

The electrochemical performance of the fabricated electrodes, in terms of charge storage kinetics, were studied through electrochemical impedance spectroscopy (EIS). EIS characterisation is associated with migration and diffusion of reactants towards or away from the electrode surface and thus, produces a peculiar frequency character known as the Warburg impedance^50^. The frequency character dependence on the impedance can demonstrate the underlying electrochemical phenomenon. EIS study was performed on the G-Ni, G-NiO, and NiO electrodes to examine, and compare the associated mechanism occurring on the electrode surface. A frequency range of 0.01–100 kHz was employed at an alternating signal of 10 mV (RMS). Figure 5 demonstrates the comparison of EIS spectra for the three electrodes. All spectra consisted of a typical semicircle, followed by a straight line. The high frequency region in the spectra gives the parametric information on the electrode resistance. The diameter of the semicircle in the spectrum is a measure of the resistance arising from the charge transfer kinetics which is associated with the electrode/electrolyte interface, and electrode geometry^51^. A straight line taken from the frequency knee point (real axis-*Z*_*re*_) depicts electrode capacitive response. Ideally, a straight line response would be parallel to the imaginary axis (*Z*_*im*_). The line gradient in the low frequency region measures the diffusion resistance called the Warburg impedance, W^12^. The illustration of the behaviour of the electrode in the high frequency region is depicted in Fig. 5 (inset) showing an expanded plot for the G-Ni, G-NiO, and NiO electrodes. As expected, the plots distinctly illustrated the semicircle in the high frequency region, and a straight line in the low frequency region.

From the Nyquist plot (inset (i) of Fig. 5), the impedance behaviour of the G-Ni electrode is portrayed and shown by a vertical line in the low frequency region showing an ideal capacitive behaviour coupled with a low charge transfer resistance (*R*_*ct*_). Similarly, the NiO electrode revealed a bigger semicircle (*R*_*ct*_ = 0.45 Ω) than G-NiO (*R*_*ct*_ = 0.16 Ω), with the least being G-Ni electrode (*R*_*ct*_ = 0.12 Ω) in the high frequency region which implied that G-Ni electrode had the lowest charge transfer resistance in comparison with others. The equivalent circuit was used to obtain the *R*_*ct*_ of the electrodes (inset (ii) of Fig. 5). Typically, three factors play a key role in the equivalent series resistance (ESR). They are; (a) discontinuity in ionic conductivity and electric conductivity occurring during charge transfer mechanism (b) resistance between NF (electron collector) and the connectors (leads), and lastly, (c) the intrinsic resistance of the NF^52^. The G-Ni electrode relatively recorded the best line which was parallel to the imaginary axis (*Z*_*im*_) and thus, proved that it had a better storage capacity due to better conductivity. This result was expected as the G-Ni electrode had a uniform thin layer of graphene well distributed on NF. However, the G-NiO electrode which comprised graphene thin film and NiO thin film in the amorphous phase was considered highly conductive and hence, the pathways for ion transport to the nickel foam were increased. This phenomenon gave rise to the high specific capacity even at high current density and making it a more appropriate material for supercapattery development.

### Asymmetric device assemblage and performance

**Figure 6:**
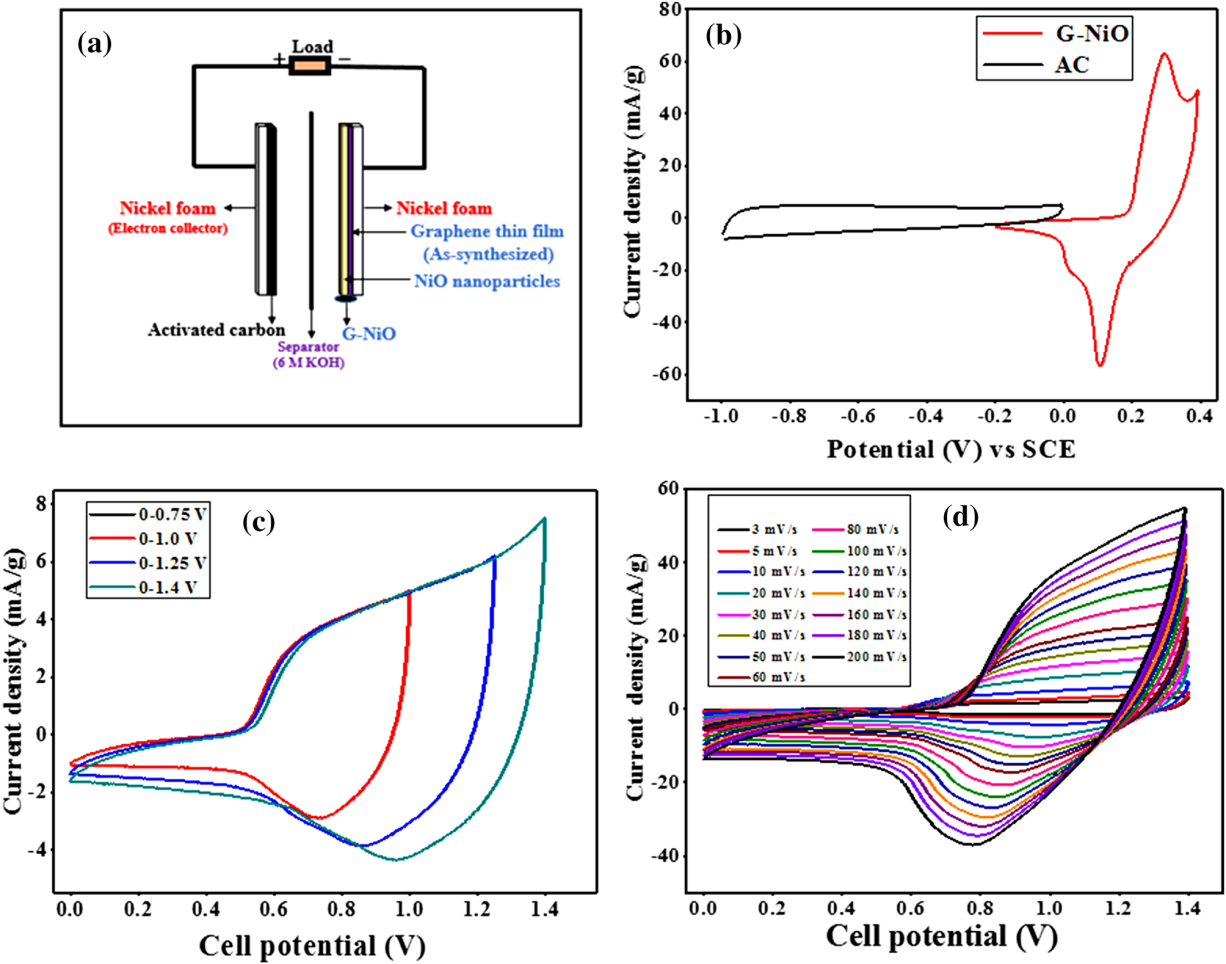
(**a**) Schematic illustration of the assembled G-NiO//AC supercapattery, (**b**) comparative CV curves of G-NiO as positive electrode and AC as negative electrode measured at a scan rate of 10 mV s^−1^ in KOH (6 M) electrolyte, (**c**) CV curve of G-NiO//AC supercapattery measured over different potential windows at a scan rate of 10 mV s^−1^, and (**d**) CV curve of G-NiO//AC supercapattery measured at diverse scan rates.

Supercapattery is a hybrid device that has the features of both capacitor and battery. The characteristic parameter in supercapattery is the energy density which is enhanced by extending the operating voltage window. Since, energy density is a function of electrode capacity and cell voltage, a supercapattery device incorporating EDLC, and battery-grade utilises the combined potential window of the two electrodes leading to a higher potential window of the device. In this study, the supercapattery was configured by employing activated charcoal (AC) as the negative electrode and G-NiO as the positive electrode as shown in Fig. 6a. As the first step in the electrochemical studies, the individual cyclic voltammograms were run for both G-NiO and AC electrodes in a three-electrode system at room temperature. These preliminary analyses allowed for the accurate estimation of the maximum possible operating potentials by investigating the individual electrode properties. It was observed that G-NiO and AC electrodes operated optimally in the potential range of − 0.2–0.4 V and − 1.0–0 V, respectively (Fig. 6b). Note that, from Fig. 6b, the potential window for the fabricated G-NiO//AC supercapattery can be extended from 0 to 1.4 V by combining the potential range of both G-NiO and AC electrodes. Thus, the maximum stable working potential window for the assembled device was taken to be 0–1.4 V. At a fixed scan rate of 10 mV s^−1^, voltammograms at different potential ranges were recorded to observe the rate capability of the device (Fig. 6c). The cyclic voltammograms of the assembled device over the potential window of 0–1.4 V recorded at diverse san rates (3–200 mV s^−1^) is represented in Fig. 6d. The capacitive carriage contribution of AC was observed in the potential range of 0–0.5 V. From Fig. 6d, it could be seen that until a potential of 0.5 V, the shape of the CV curve was rectangular which manifested that the charge storage was mainly due to the EDLC effect. However, at potentials beyond (above 0.5 V), redox peaks also appeared revealing that charge storage was due to the Nernstian processes. Hence, over the full potential window of 0 to 1.4 V, the charge storage mechanism was due to both, EDLC and non-capacitive faradaic reactions. At the potential range of 0–0.5 V, energy storage was contributed by capacitive behaviour while beyond 0.5 V (0.5–1.4 V), non-capacitive faradaic redox reactions were dominant in the energy storage mechanism. High rate capability and stability of the supercapattery had been demonstrated by the shape, constancy, and amplification of the CV curves at the various scan rates (3–200 mV s^−1^).

**Figure 7:**
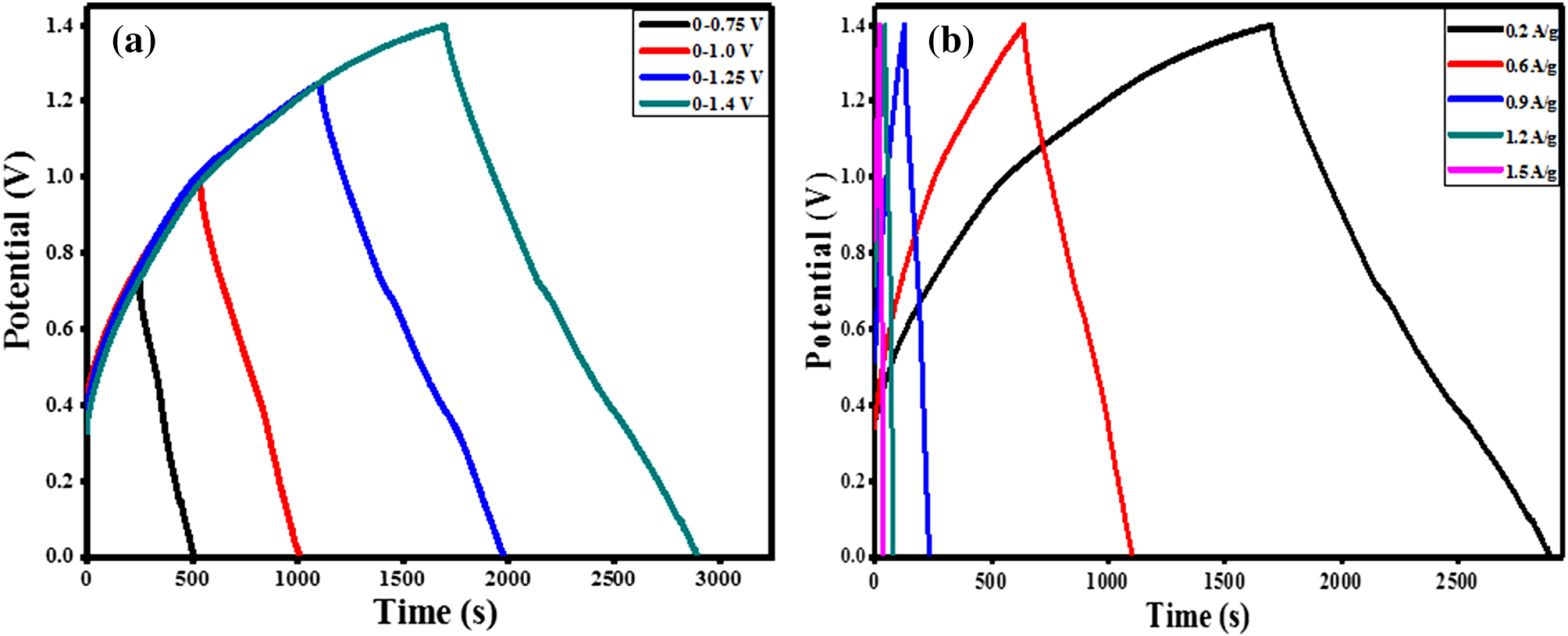
GCD plots of G-NiO//AC supercapattery measured at (**a**) different potential windows, and (**b**) different current densities.

An estimation of the specific capacity, *Q*_*d*_ of the assembled asymmetric device was done using the relation in Eq. (3). The assembled asymmetric device operated at a specific capacity of 67.8 mA h g^−1^ at a current density of 0.2 A g^−1^. Table S3 presents the specific capacity values against the diverse current densities of the device while Fig. S1 illustrates the device rate capacity (see supplementary data online). Figure 7a depicts the charge–discharge plots of the G-NiO//AC supercapattery (asymmetric device) measured over different potential window at the same current density to observe the device performance while Fig. 7b shows the charge–discharge plots at various current densities in the potential range of 0–1.4 V. The symmetrical charge–discharge plots illustrated the capacitive nature combined with high reversible redox reactions. To evaluate the efficiency of the assembled device, the energy density, *E *(W h kg^−1^), and power density, *P *(W kg^−1^) are the key parametric quantities of the fabricated supercapattery and therefore, were calculated by using the relation in Eqs. (4) and (5), respectively.

3 $$Q_d=\frac{{I \times \Delta t}}{{3.6 \times m}}$$

4 $$E=\frac{{{Q_s} \times \Delta V}}{{2 \times 3.6}}$$

5 $$P=\frac{{E \times 3600}}{{\Delta t}}$$

where *Q*_*d*_ is the specific capacity of the device (C g^−1^), $\Delta V$ is the operating voltage window *(V),* and $\Delta t$ is the discharge time (s). The specific energy and power densities were measured as 47.3 W h kg^−1^ and 240 W kg^−1^ respectively. The results of the assembled supercapattery are comparable with some published studies that employed battery-grade materials as positrode such as MnCo_2_O_4_//AC (33.8 W h kg^−1^ at 318.9 W kg^−1^)^51^, CONF//AC (23.7 W h kg^−1^ at 307 W kg^−1^)^50^, MWCNT-Co_3_O_4_-Ag//AC (16.5 W h kg^−1^ at 297.5 W kg^−1^)^12^, rGO-Co_3_O_4_-Ag//AC (23.63 W h kg^−1^ at 440 W kg^−1^)^13^, and PbO_2_//AC (27 W h kg^−1^ at 152 W kg^−1^)^53^.

**Figure 8:**
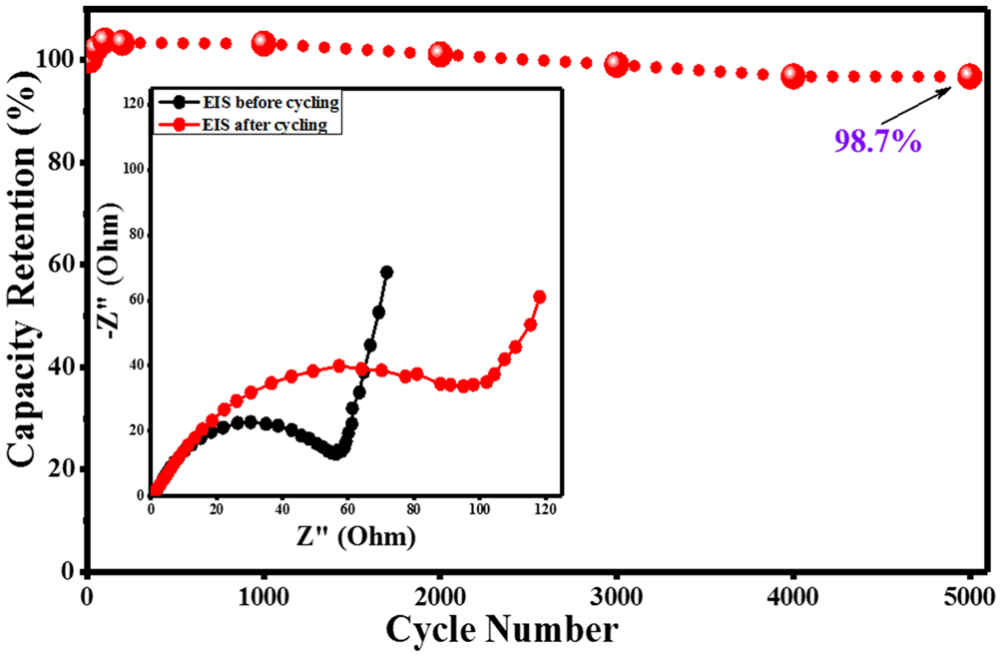
Cyclic stability of G-NiO//AC supercapattery; inset shows the comparative EIS spectra of the device showing the internal resistances before and after cycling.

To evaluate the viability of the assembled supercapattery employing G-NiO as the positrode, a prolonged cycling stability studies were performed over charge–discharge of 5,000 cycles. Figure 8 represents the cyclic stability performance of the assembled asymmetric device showing 98.7% specific capacity retention after 5,000 cycles. From the stability test, it could be observed that the activation process of the electrodes in the electrolyte was stable. Initially, the specific capacity increased until 100 cycles, and then decreased slightly (≈ 1%) until 500 cycles, and then gradually and slowly decreased until the 5,000th cycle. Fortunately, 97.8% capacity retention was achieved after 5,000 cycles for the G-NiO//AC supercapattery. The high cyclability of the assembled supercapattery using G-NiO electrode assures its viability for practical usage. The charge–discharge plot for the first 10 cycles (during cycling) is shown in the supplementary data online (Fig. S6).

Figure 8 (inset) depicts the overlay of the Nyquist plot for the assembled device showing the EIS spectra of initial (before) and after life cycle test, and portrays the cyclic stability of the device. As expected, in the high frequency region, a semicircle was apparent whereas, in the low frequency region, a straight line was observed for the device. The semicircle diameter measurements denoted the low cell resistance before cycling which suggested a short path travelled by an ion/electron. However, after cycling, the EIS spectrum revealed that the ion/electron transport pathway was longer as indicated by the higher charge transfer resistance. Similarly, before cycling, the low frequency region of the plot showed the high line gradient in the Nyquist plot indicating low interfacial diffusion resistance (fast ion diffusion and mass transport at electrode/electrolyte interface). Accordingly, the Nyquist plots reported the charge transfer resistances of the device to be *R*_*ct*_ = 0.175 Ω (before) and *R*_*ct*_ = 0.182 Ω (after life cycle test). As an investigation of the morphological properties of the G-NiO electrode after cycling, a post-mortem analysis was done through FESEM to observe the morphological changes after the life cycle test. The micrographs for the freshly fabricated G-NiO is depicted in Fig. S7 while the micrograph showing the structural and morphological degradation of the electrode after the life cycle test is shown in Fig. S8 (see supplementary data online).

## Conclusions

A three-dimensional (3D) graphene electrodeposited nickel oxide thin film (G-NiO) nanocomposite as a binder-free electrode has been successfully fabricated by a two-step route: APCVD and electrochemical deposition. The binary binder-free nanocomposite exhibited an excellent electrochemical performance owing to the synergistic effect of NiO thin film, and a highly conductive graphene platform. Moreover, the fabricated supercapattery in the configuration of G-NiO//activated charcoal supercapattery demonstrated a remarkable electrochemical performance. The assembled supercapattery (G-NiO//AC) portrayed a good balance between the parametric quantities of the device in terms of its energy density and power density. Furthermore, the evaluation of the device cyclic stability showed an excellent cycle life with only a 1.3% loss of its initial specific capacitance after 5,000 cycles. Hence, the unique properties of the supercapattery characterize its excellent electrochemical performance and thus, ensures sustainability.

## Experimental

### Materials

Nickel foam (NF) of uniform thickness − 1.6 mm, bulk density—0.45 g cm^−3^, porosity—95% (GF28024657) was supplied by Sigma Aldrich, Malaysia. Activated charcoal (AC), Nickel (II) chloride hexahydrate (NiCl_2_·6H_2_O), Potassium hydroxide (KOH), Hydrochloric acid (HCl-37%), ethanol (C_2_H_6_O-95%), and acetone (CH_3_H_6_O-99.5%) were supplied by NA One Solution, Malaysia. Analytical grades of hydrogen (H_2_-99.99% purity), methane (CH_4_-99.995% purity), and Argon (Ar-99% purity) gases were supplied by MOX Sdn. Bhd, Malaysia. All the reagents were used as received without further processing and de-ionized (DI) water was used throughout the experiment.

### Characterisation techniques

The experimental results validation was done using various characterization techniques. The quality of the graphene (in terms of purity) as well as the properties of the G-NiO electrode were investigated using the WITec’s ALPHA 300 M+ at an excitation wavelength of 514 nm (Raman spectroscopy). The surface morphology, elemental composition, and mappings were studied using a JEOL: JSM-7800F microscope fitted with energy dispersive X-ray analyser (FESEM-EDS). The crystalline nature and phase identification of the fabricated electrodes were recorded using a PANalytical-X’Pert MPD X-ray diffractometer equipped with Cu K-α radiation (λ = 1.5418 Å) at a scan rate of 0.2 s^−1^; step 0.05° over a 2θ range of 5° to 90°.

### Electrochemical (EC) measurements

The electrochemical workstation, VersaSTAT-3F model, was used to examine the electrochemical performance of the fabricated electrodes at room temperature. A 6 M KOH, saturated calomel electrode (SCE), and a platinum wire were used as the electrolyte, reference, and counter electrodes, respectively. The procedure involved the dipping of the sample electrode, with a dimension of 1 × 1 cm (working electrode) into the aqueous electrolyte. Cyclic voltammetry (CV), galvanostatic charge–discharge (GCD), and electrochemical impedance spectroscopy (EIS) techniques were used to determine the electrochemical parameters of the electrodes. The CV voltammograms were recorded within certain potential windows at different scan rates, while the GCD characteristics were measured at different potential windows, and various current densities. The EIS measurements were recorded in the frequency range of 0.01 Hz to 100 kHz at alternating current voltage of 10 mV (RMS).

### Fabrication of 3D binder-free graphene electrode

**Figure 9:**
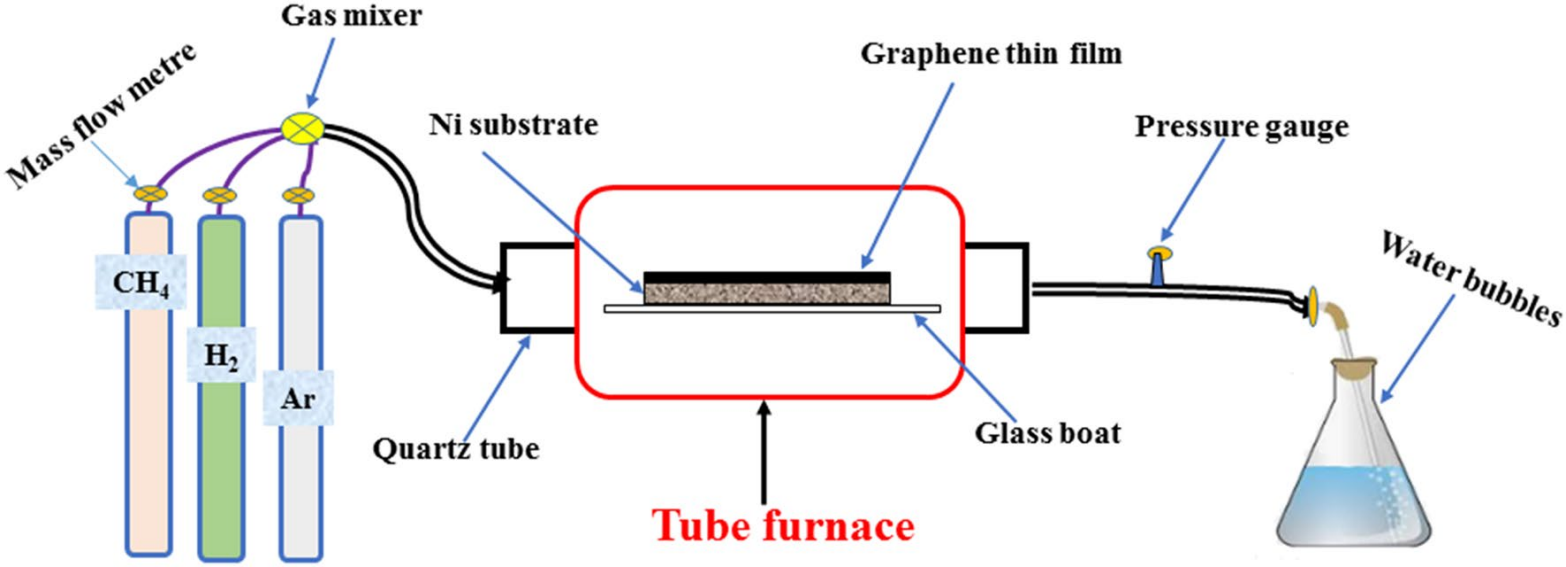
Schematic illustration for the APCVD of G-Ni electrode.

Firstly, pre-treatment of the nickel substrate was done using HCl, DI water, and ethanol. HCl (0.1 M) was used to wash the substrate—NF (size: 1 cm × 2 cm) under ultrasonic bath for 10 min to remove the native oxide layer and dirt. The substrate was further cleaned with DI water in the bath sonicator for 10 min at room temperature. Finally, the substrate was rinsed with ethanol and dried in a vacuum oven at 80 °C for 6 h. The pre-treated substrate was then placed in a clean glass boat and loaded into a tube furnace. Atmospheric pressure CVD was used to grow graphene on the substrate under optimal synthesis conditions. The nickel foam served two purposes in the synthesis; firstly, as a catalyst in lowering the activation energy of the gaseous precursor and secondly, as a support material for the growth of graphene thin film. Methane (CH_4_) was employed as the precursor gas. Hydrogen (H_2_) was used as the etching gas, while Argon (Ar) was used to de-oxygenate the furnace and to maintain an uncontaminated synthesis environment throughout the synthesis period. Figure 9 depicts is the experimental setup for the preparation of the 3D graphene electrode (G-Ni).

### Fabrication of 3D graphene supported NiO electrode

A solution of NiCl_2_·6H_2_O (0.50 M) was prepared which served as the electrolyte and precursor for the growth of NiO thin film onto the G-Ni electrode. The electrodeposition was carried out in a three-electrode cell configuration in which the G-Ni electrode was designated as the working electrode, while platinum wire and SCE served as the counter and reference electrodes, respectively. Electrodeposition was conducted through chronoamperometry for 20 min at a constant potential of −  1.2 V to grow NiO onto the G-Ni electrode. After electrodeposition, the working electrode was removed, washed with DI water, and dried in an oven at 80 °C for 12 h. Then, it was re-weighed to determine the mass loading of NiO on the G-Ni electrode. The fabricated nanocomposite was then designated as the G-NiO electrode.

## Supplementary information

Supplementary file1 (PDF 324 kb)
