# Supplementary material for: Fabrication of 3D binder-free graphene NiO electrode for highly stable supercapattery
Source: Sci Rep. 2020 Jul 8;10:11214. doi: 10.1038/s41598-020-68067-2 (PMC7343816; doi:10.1038/s41598-020-68067-2)
Supplement: Supplementary file 1 — Supplementary file1 (PDF 324 kb) [file 41598_2020_68067_MOESM1_ESM.pdf]

Fabrication of 3D Binder-Free Graphene NiO Electrode for Highly Stable Supercapattery

Elochukwu Stephen Agudosi[1], Ezzat Chan Abdullah[1,*], Arshid Numan[2,6], Nabisab Mujawar Mubarak[3,*], Siti Rahmah Aid[4,7], Raul Benages Vilau[5], Pedro Gómez-Romero[5], Mohammad Khalid[6,*] & Nurizan Omar[1]

***Corresponding authors**
Email address: ezzatc@utm.my (E.C. Abdullah), mubarak.yaseen@gmail.com or mubarak.mujawar@curtin.edu.my (N.M. Mubarak), khalids@sunway.edu.my (M. Khalid)

Supplementary information S1:

Table S1: Specific capacity values against scan rate

Electrode material	Scan rate (mV/s)	Specific capacity (C/g)
NF (reference)	3	0.86
G-Ni (reference)	3	49.66
NiO	3	96.03
	5	82.53
	10	58.74
	20	36.15
	30	25.46
	40	16.15
	50	12.54
G-NiO	3	242.68
	5	190.38
	10	133.68
	20	83.99
	30	56.40
	40	44.97
	50	35.73

Table S2: Specific capacity values against current density

Electrode material	Current density (A/g)	Specific capacity (C/g)
(a) NiO	0.6	43.60
	0.8	22.40
	1	20.84
	2	18.0
(b) G-NiO	0.6	91.80
	0.8	32.0
	1	28.61
	2	26.40

Table S3: Specific capacity values of device against current density

Device	Current density (A/g)	Specific discharge rate (mAh/g)
G-NiO//AC	0.2	67.8
	0.6	49.02
	0.9	39.45
	1.2	21.04
	1.5	15.31

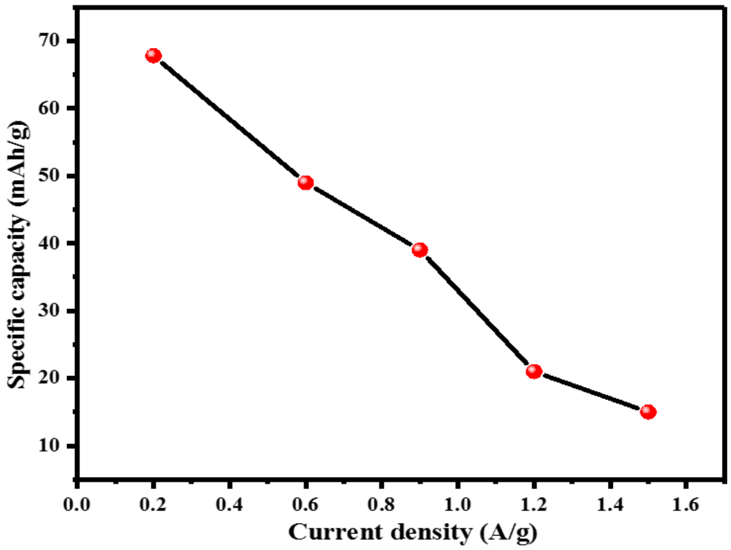

Fig. S1: Plot of specific capacity against current density showing the rate capacity of the device

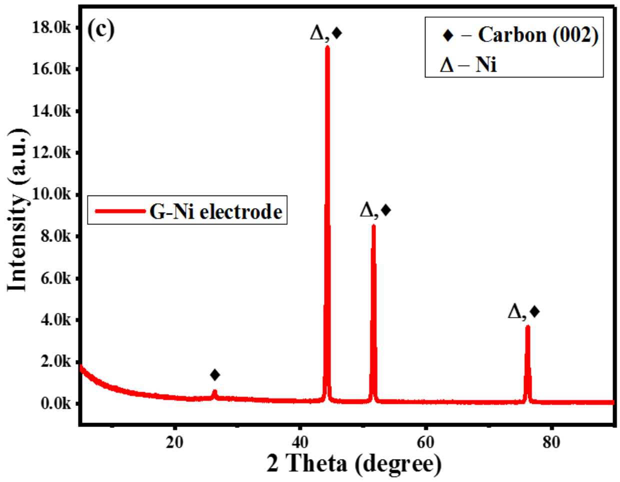

Fig. S2: XRD spectrum of G-Ni electrode showing the carbon peak with lattice parameter (002)

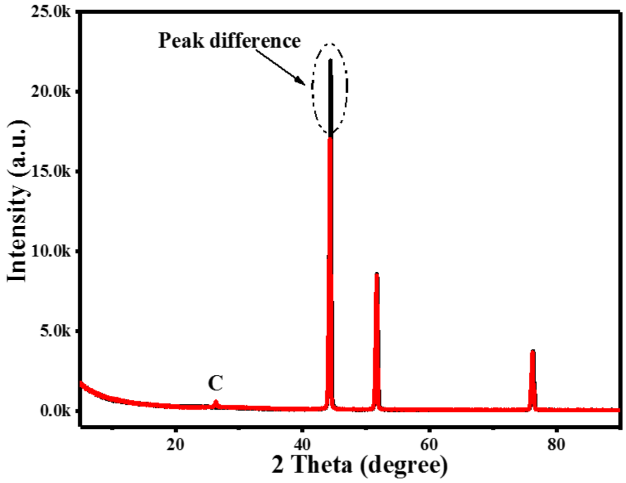

Fig. S3: Overlay of XRD patterns of NF and G-Ni electrode showing the difference in peak intensities.

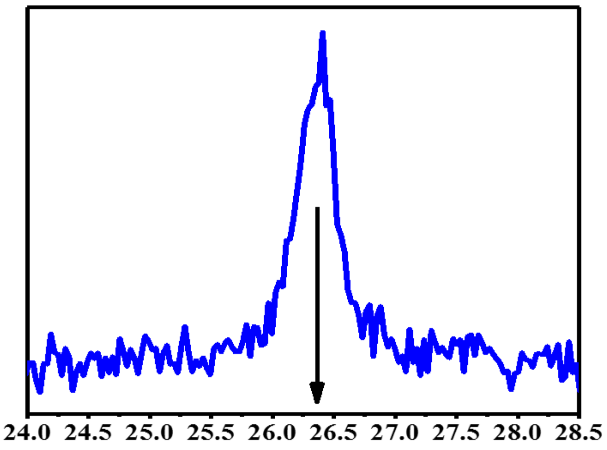

Fig. S4: XRD pattern for graphene showing the exact 2 theta degrees peak position

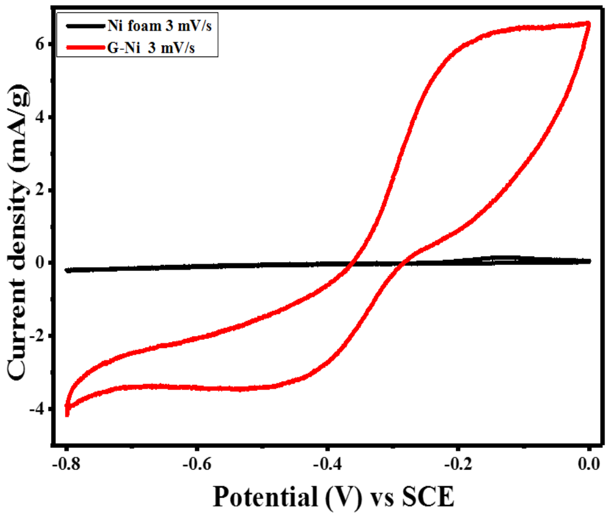

Fig. S5: Overlay of CV curves of bare Ni foam and G-Ni electrode

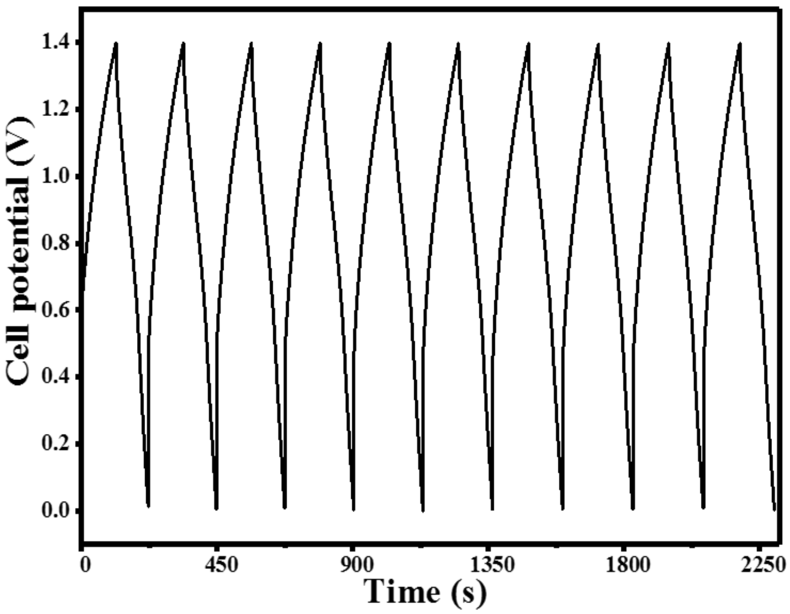

Fig. S6: A plot of the first 10 cycles of the charge-discharge for device stability test

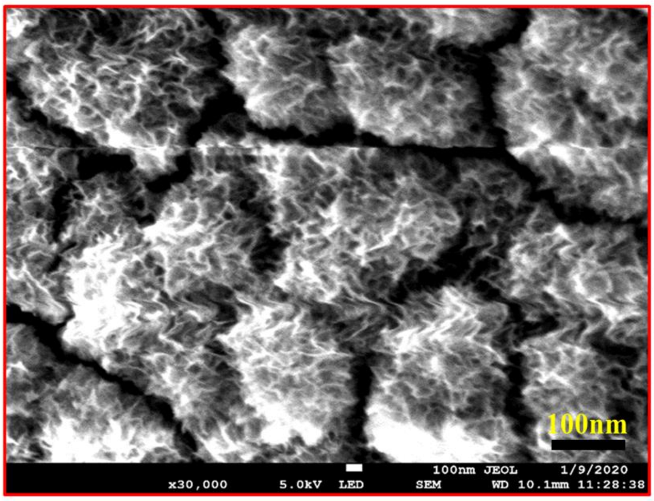

Fig. S7: FESEM image of G-NiO electrode before cycling

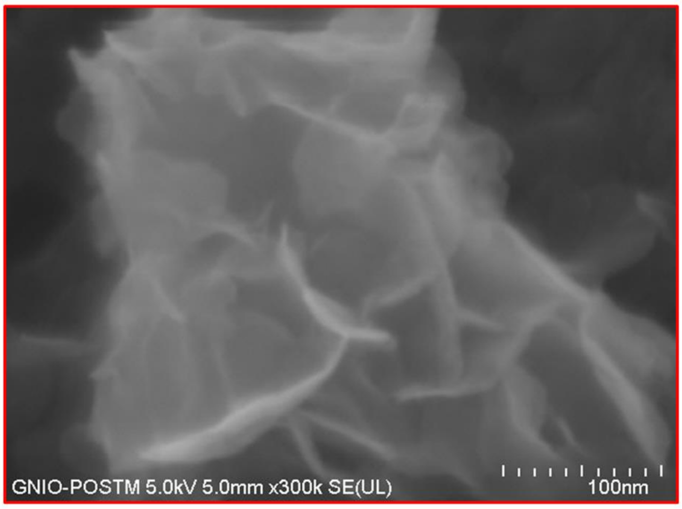

Fig. S8: FESEM image of G-NiO electrode after cycling (Post mortem)

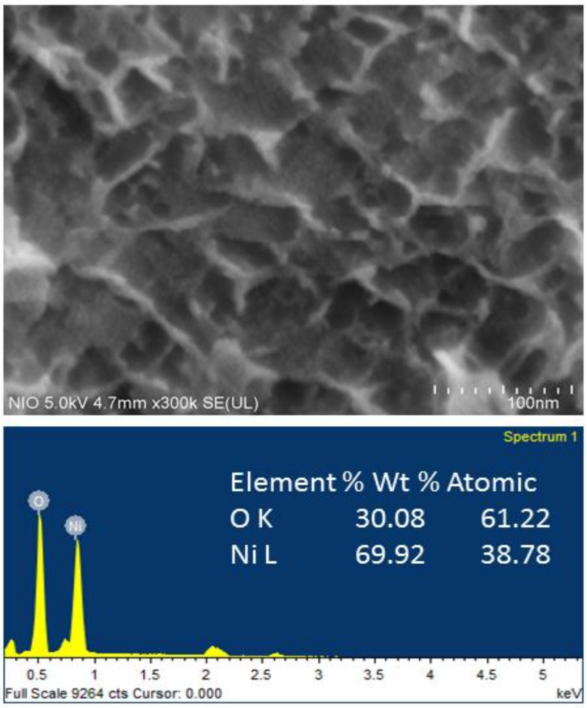

Fig. S9: FESEM image and EDS spectrum of NiO electrode
